# AgBR1 antibodies delay lethal *Aedes aegypti*-borne West Nile virus infection in mice

**DOI:** 10.1038/s41541-019-0120-x

**Published:** 2019-07-08

**Authors:** Ryuta Uraki, Andrew K. Hastings, Doug E. Brackney, Philip M. Armstrong, Erol Fikrig

**Affiliations:** 1 The Anlyan Center for Medical Research and Education, Department of Internal Medicine, Section of Infectious Diseases, Yale University School of Medicine, 300 Cedar Street, New Haven, CT 06520-8031 USA; 2 Center for Vector Biology and Zoonotic Diseases, Department of Environmental Sciences, The Connecticut Agricultural Experiment Station, New Haven, 06511 CT USA; 3 Howard Hughes Medical Institute, Chevy Chase, MD 20815 USA

**Keywords:** Vaccines, West nile virus, Viral infection

## Abstract

West Nile virus (WNV) is transmitted by mosquitoes and can cause severe disease, including meningoencephalitis. AgBR1 is a mosquito salivary protein that enhances *Aedes aegypti* mosquito-borne Zika virus pathogenesis in mice. Here, we show that AgBR1 antibodies reduce the initial West Nile viral load and delay lethal infection after feeding by an infected *Aedes aegypti* mosquito. Targeting AgBR1 may therefore be incorporated into strategies to prevent mosquito-transmitted West Nile virus infection.

## Introduction

West Nile virus (WNV) is a single-stranded positive-sense RNA virus in the genus *Flavivirus*, which normally circulates in a bird-mosquito transmission cycle and is a common human mosquito-borne flaviviral infection in North America and other regions in the world.^1–3^ WNV can also infect horses, and other non-avian vertebrate hosts.^4^ Despite substantial efforts, effective FDA-approved preventive or therapeutic measures are not yet available.^1,4,5^

*Culex* mosquito *spp*., now endemic in tropical and subtropical regions as well as more temperate areas, are the major vectors for WNV worldwide.^5^ However, the virus also has been isolated from *Aedes aegypti* mosquitoes, which is present in tropical and subtropical locations as well^6,7^ and are a potential threat for transmission of WNV to humans.^8^ Although the vector competence of WNV in *Ae. aegypti* is lower than that of *Culex* spp., multiple factors can affect vector distribution, including climate change, and this may influence the vectorial capacity of *Aedes* mosquitoes for WNV in the future.^9,10^ Moreover, in laboratory studies, *Ae. aegypti* readily feed on mice and a well-annotated whole genome sequence is available.^11^

When mosquitoes take a blood meal, they inoculate saliva into the skin.^12^ Mosquito saliva contains molecules which modulate various host responses, including coagulation, platelet aggregation, thrombin activation, vasodilation, and other mammalian host pathways.^13,14^ Previous studies, including some from our group, demonstrate that components of saliva enhance the pathogenicity and transmission of arboviruses including WNV, dengue, Zika, and Semliki Forest viruses, suggesting that certain salivary proteins are important for influencing flavivirus infectivity during transmission from vector to host.^15–19^ Recently, we identified *Ae. aegypti* AgBR1 as an antigenic protein in vertebrate hosts fed upon by *Ae. aegypti* mosquitoes.^20^ The expression of AgBR1 in the salivary glands is up-regulated after blood feeding and AgBR1 belongs to a family of proteins that have lost chitinolytic activity,^21,22^ however, the function of this protein in the vertebrate host remains unclear. We showed that *Ae. aegypti* AgBR1 modulates the early immune response in the murine skin following mosquito bite and that immunization against AgBR1 partially protects mice from a lethal mosquito-borne Zika virus infection.^20^ To determine whether this effect extends beyond Zika virus to another flavivirus, we examined the influence of AgBR1 antibodies against *Ae. aegypti*-borne WNV infection in mice.

## Results and discussion

**Fig. 1:**
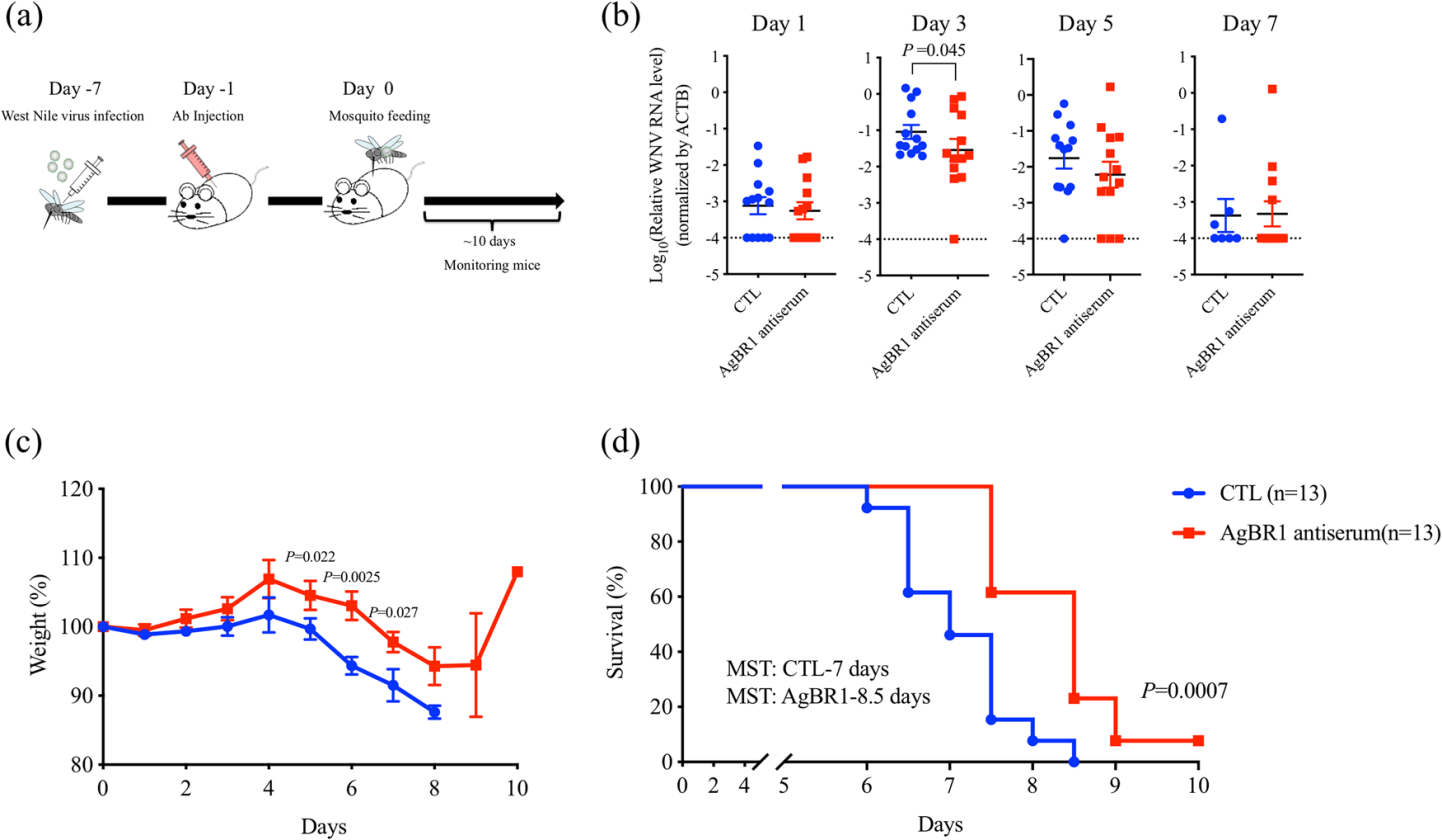
AgBR1 antiserum protects mice from mosquito-borne WNV virus infection. **a** The schematic of the experiment. Mice were administrated AgBR1 antiserum one day before WNV-infected mosquito feeding. Immunized mice were monitored for survival for 10 days after infected mosquito feeding. **b** The virus levels in blood of mice fed by an infected mosquito. Blood was collected every other day for 7 days from mice fed on by WNV-infected mosquitoes and analyzed by qRT-PCR. WNV RNA levels were normalized to mouse *β* actin RNA levels. Mice immunized with naïve serum served as controls. Error bars represent mean ± SEM. Each data point represents one mouse. Normalized viral RNA levels were analyzed using one-tailed Wilcoxon–Mann–Whitney test (*n* = 13/each group biologically independent samples pooled from three separate experiments). **c** The weight of mice fed by an infected mosquito. Mice were monitored daily after WNV infection. Error bars represent mean ± SEM. Weight at each time point were compared using one-tailed Wilcoxon–Mann–Whitney test (*n* = 13/each group biologically independent samples pooled from three separate experiments). **d** Survival was assessed by a Gehan–Wilcoxon test (*n* = 13/each group biologically independent samples pooled from three separate experiments)

To determine whether targeting AgBR1 altered pathogenesis during mosquito-borne WNV infection, we passively immunized mice with AgBR1 antiserum before challenging them with WNV by mosquito bite. *Ae. aegypti* mosquitoes were used as a vector model, since the well-annotated whole genome sequence and easy maintenance make this species ideal for laboratory viral transmission studies.^11,23^ Wild type Swiss Webster mice were administered AgBR1 or control antiserum and 24 h later were bitten by WNV-infected *Ae. aegypti* mosquitoes (Fig. 1a). Passive immunization with AgBR1 antiserum significantly reduced WNV RNA levels in the murine bloodstream at an early stage (3 days) of infection (Fig. 1b). Our group and others have previously demonstrated that components of mosquito saliva modulate local host responses and recruit several immune cells which can be target of virus replication.^17,20,24^ This may lead to virus dissemination at an earlier, rather than later, time point. Although we were not able to detect a significant difference at day 1, it may be due to the complex interplay of recruited immune cells at the bite site, which leads to shifting populations of WNV-susceptible cells over the first hours and days of infection. In addition, pretreatment with AgBR1 antiserum delayed virally-induced weight loss (Fig. 1c) and prolonged median survival time of mice by 20% (Fig. 1d). As the mosquito-borne WNV infection model used in this study (survival rate: 0%, median survival time: 7 days in control) is much more virulent than our previous mosquito-borne Zika infection model (survival rate: 30–45%, mean survival time: 12–25 days in control),^20^ the 1.5-day delay of fatal outcome is noteworthy. Overall, our results indicate that blocking AgBR1 suppresses virus replication and/or dissemination at early time points and alters mosquito-borne WNV infection.

We then determined whether the effects observed with AgBR1 were specific to this protein or if any antigenic *Ae. aegypti* salivary gland protein was capable of affecting WNV infection. We chose an additional protein, the putative 34 kDa family secreted salivary protein (SP), which was the top hit from our previous yeast surface display screen. Sera from mice bitten by mosquitoes showed strong reactivity to the SP protein.^20^ SP has also been reported as a salivary gland protein that was upregulated during flavivirus infection.^18^ We performed identical passive immunization experiments using SP antiserum. SP antiserum did not alter viremia, weight loss or survival time after lethal mosquito-borne WNV infection (Supplementary Fig. 1).

**Fig. 2:**
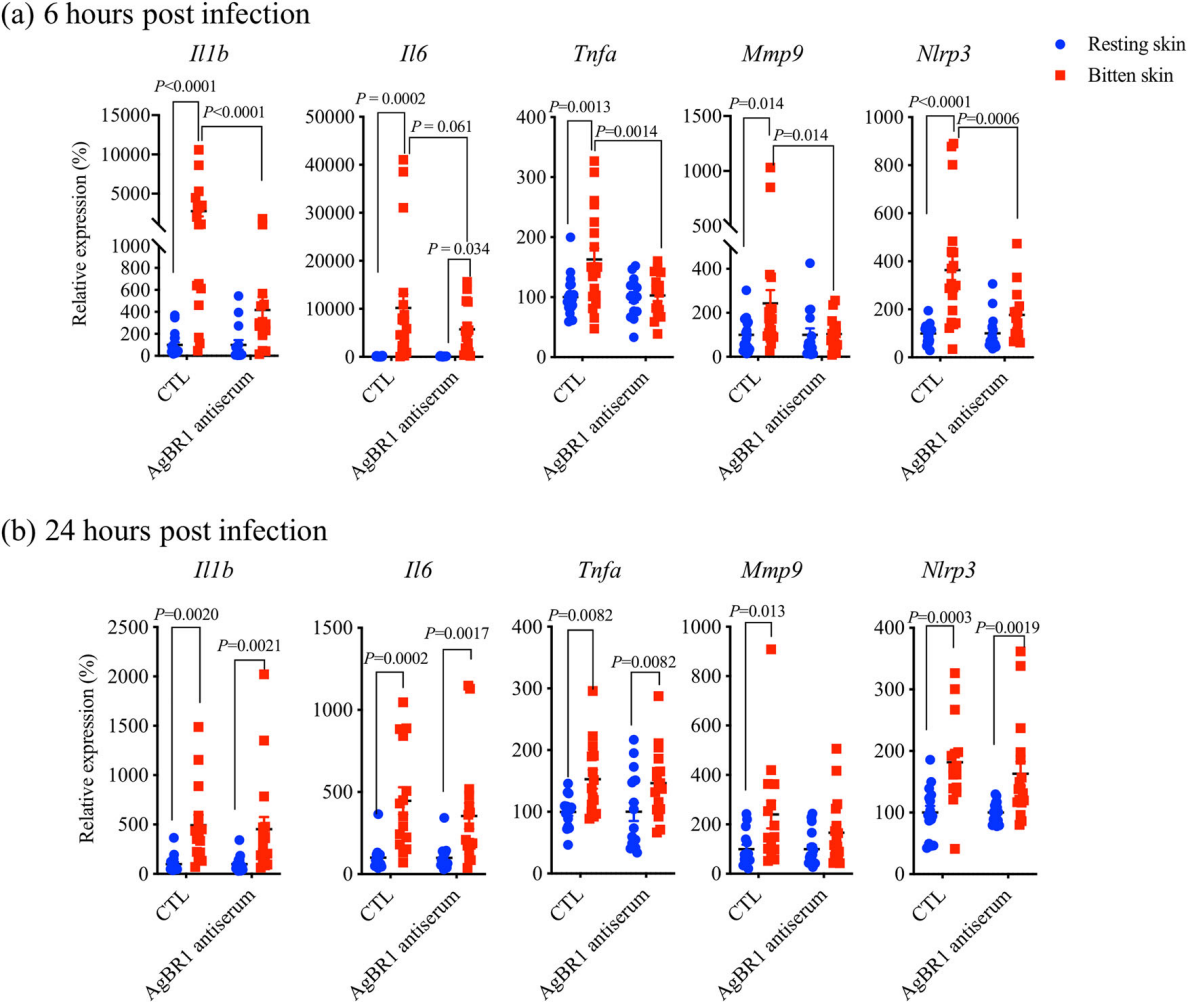
AgBR1 antiserum modulates host responses at the WNV-infected mosquito bite site. The expression levels of several cytokines were analyzed by qRT-PCR at **a** 6 h or **b** 24 h after bites of infected mosquitoes, which is normalized to mouse *β* actin RNA levels. Error bars represent mean ± SEM. Each dot represents one bitten or control site. Significance was determined by two-way ANOVA test (6 h; *n* = 19/control group, *n* = 15/AgBR1 antiserum group, 24 h; *n* = 15/control group, *n* = 17/AgBR1 antiserum group biologically independent samples pooled from two separate experiments)

Our previous study showed that AgBR1 antiserum suppresses early host responses at bitten sites after Zika virus-infected mosquito feedings in AG129 mice.^20^ Therefore, we examined whether AgBR1 antibodies alter the early host responses after feeding by mosquitoes infected with WNV. Proinflammatory genes including *Il1b*, *Il6*, and *Tnfα*, were significantly suppressed at the bite site in mice treated with AgBR1 antiserum at 6 h post feeding (Fig. 2a). In addition to these genes, we examined the expression levels of *Mmp9*, which is previously reported to play an important role in WNV entry into the brain,^25^ and those of *Nlrp3*, which is a key molecule of NLRP3 inflammasome that drives IL-1β signaling and is involved in WNV control in the central nervous system (CNS).^26^ As shown in Fig. 2a, *Mmp9 and Nlrp3* genes were significantly suppressed in AgBR1 antiserum-treated mice at 6 h post feedings. Interestingly, we did not see differences in the expression level of any of these genes 24 h after bites with WNV-infected mosquitoes (Fig. 2b). In our previous study of AgBR1 and Zika virus, we observed significant suppression of several proinflammatory genes and cytokines at 24 h post Zika virus-infected mosquitoes, and there are a few underlying differences in the experimental design that could explain these differences. One major difference is that the current study of WNV took advantage of immunocompetent wild type mice, as contrasted with the immuno-incompetent *Ifnar1*^*−/*−^ *Ifngr*^*−/−*^ mice used for the Zika virus study. Therefore, it is possible that the difference of mouse models could cause the time lag of early host responses. Overall, these results demonstrated that AgBR1 antiserum suppresses the early local host responses after WNV-infected mosquito feedings, suggesting that the suppression of host responses by AgBR1 antibodies leads to the delay of viral dissemination and fatal outcome.

AgBR1 antiserum also specifically recognizes a protein in *Culex pipiens* salivary glands (Suplementary Fig. 2). Since *Ae. aegypti* AgBR1 has high homology (amino acid identities = 80%) with *Culex* spp. chitotriosidase-1 protein, which is predicted in silico to be secreted from the salivary gland, we hypothesize that this *Culex* protein recognized by AgBR1 antiserum may have similar function during WNV infection. Further work will be needed to examine whether immunization against the *Culex* homologous protein has a protective effect in WNV infection transmitted by *Culex* mosquitoes.

In conclusion, our study demonstrated that passive immunization with AgBR1 antiserum delays lethal *Ae. aegypti*-borne WNV infection in mice, as we have previously shown for Zika virus infection transmitted by the same mosquitoes. Therefore, given the results of this and other studies, we suggest that the strategy of targeting individual arthropod salivary factors such as AgBR1 might be broadly applicable to other mosquito-borne pathogens.

## Methods

### Ethics statement

All experiments were performed in accordance with guidelines from the Guide for the Care and Use of Laboratory Animals of the NIH. The animal experimental protocols were approved by the Institutional Animal Care and Use Committee (IACUC) at the Yale University School of Medicine. Procedures for handling and care of animals were approved by and performed under the Animal Care and Use Committee at The Connecticut Agricultural Experiment Station. All infection experiments were performed in a biosafety level 3 animal facility, according to Yale University and the Connecticut Agricultural Experiment Station regulations. Every effort was made to minimize murine pain and distress. Mice were anesthetized with ketamine/xylazine for mosquito infection experiments and euthanized as suggested by the IACUC.

### Mosquitoes and animals

*Ae. aegypti* (Orlando strain, collected from Orlando, FL in 1952) and *Cx*. *pipiens* mosquitoes were maintained on 10% sucrose feeders inside a 12″ × 12″ × 12″ metal mesh cage (BioQuip #1450B) at 28 °C and ~80% humidity. Eggs were generated via blood meal feeding on an artificial membrane feeder with defibrinated sheep’s blood (Hemostat Laboratories). All mosquitoes were housed in a warm chamber in a space approved for BSL3 and ACL3 research. Four-week-old male mice (Swiss Webster mice from Charles River) were used in the WNV infection studies. All mice were kept in a pathogen-free facility at the Connecticut Agricultural Experiment Station.

### Mosquito injection and dissections

For WNV injection, WNV-filled needles were carefully inserted into the thorax of each mosquito and 69 nl of virus (3.4 × 10^3^ PFU) was injected using a Nanoject II auto-nanoliter injector (Drummond). Infected mosquitoes were placed back in paper cups with mesh lids and maintained in triple containment for 7 days in a warm chamber. After feeding infected mosquitoes on naïve mice, they were knocked-down on ice and salivary glands were dissected to examine the virus levels.

### Passive immunization studies

Mice were injected intraperitoneally with 150 µl per animal of AgBR1 or SP antiserum or naive rabbit serum one day before challenge. On the following day, mice were anesthetized with ketamine–xylazine and fed on by a single WNV-infected mosquito per mouse. The blood of fed mice was collected at 1, 3, 5, and 7 days post infection. Survivals and weights were monitored every day. Mice exhibiting weight loss of >20% of initial body weight or neurologic disease were euthanized. Viremia levels were examined at 1, 3, 5, and 7 days post infection by quantitative real time-PCR.

### Analysis of local immune responses after bites of West Nile virus-infected mosquitoes

Mice were passively immunized with either AgBR1 or naive antiserum 24 h prior to allowing infected *Ae. aegypti* mosquitoes to feed on the left ear. After 6 or 24 h post feedings, mice were euthanized, and the locations bitten by mosquitoes and naïve locations on the opposite ear were punched using a Disposable Biopsy Punch. Total RNA was extracted by the RNeasy Fibrous Tissue Mini Kit (QIAGEN) according to the manufacturer’s instructions. For quantitative RT-PCR, the cDNA was generated with iScript cDNA Synthesis Kit (Bio-rad) according to manufacturer’s protocol. Gene expression was examined by qRT-PCR using IQ™ SYBR Green Supermix. Target gene mRNA levels were normalized to mouse *β* actin RNA levels according to the 2^−ΔΔCt^ calculations. The qRT-PCR primer sequences are available upon request.

### Immunoblot

Three sets of salivary glands from *Ae. aegypti* and *Cx. pipiens* were placed in 20 μl Novex 2x Tris-Glycine SDS Sample Buffer, heated to 85 °C for 5 min, diluted 1:1 with water and the whole sample was loaded on a 16% Tris-glycine gel. AgBR1 and homologous proteins were examined with AgBR1 antiserum (1:1000 dilution), followed by incubation with HRP-conjugated secondary antibodies.

### Statistical analysis

GraphPad Prism software was used to analyze all the data. Mouse β actin-normalized viral RNA levels and body weights were analyzed using the Wilcoxon–Mann–Whitney test. Host responses in vivo was performed using a two-way ANOVA for multiple comparisons. Survival was assessed by a Gehan–Wilcoxon test. A *p* value of <0.05 was considered statistically significant.

### Reporting summary

Further information on experimental design is available in the Nature Research Reporting Summary linked to this article.

## Supplementary information

Supplemental Information

Reporting Summary

## Data Availability

The data that support the findings of this study are available from the corresponding authors upon request.

## Supplementary information

**Supplementary information** accompanies the paper on the *npj Vaccines* website (10.1038/s41541-019-0120-x).
