# Supplementary material for: AgBR1 antibodies delay lethal Aedes aegypti-borne West Nile virus infection in mice
Source: NPJ Vaccines. 2019 Jul 8;4:23. doi: 10.1038/s41541-019-0120-x (PMC6614468; doi:10.1038/s41541-019-0120-x)
Supplement: Supplementary file 1 — Supplemental Information [file 41541_2019_120_MOESM1_ESM.pdf]

AgBR1 antibodies delay lethal *Aedes aegypti*-borne West Nile virus infection in *mice*

Ryuta Uraki [1], Andrew K. Hastings [1], Doug E. Brackney [2], Philip M. Armstrong [2], Erol Fikrig [1, 3*]

[1] Section of Infectious Diseases, Department of Internal Medicine, Yale University School of Medicine, New Haven, CT, 06520, USA.
[2] Center for Vector Biology and Zoonotic Diseases, Department of Environmental Sciences, The Connecticut Agricultural Experiment Station, New Haven, 06511, CT, USA
[3] Howard Hughes Medical Institute, Chevy Chase, MD, 20815, USA.

*Correspondence: Erol Fikrig, Section of Infectious Diseases, Department of Internal Medicine. Yale University School of Medicine, The Anlyan Center for Medical Research and Education, 300 Cedar Street, New Haven, CT 06520-8031. Phone: (203) 785-4140, email: erol.fikrig@yale.edu

Supplementary Information

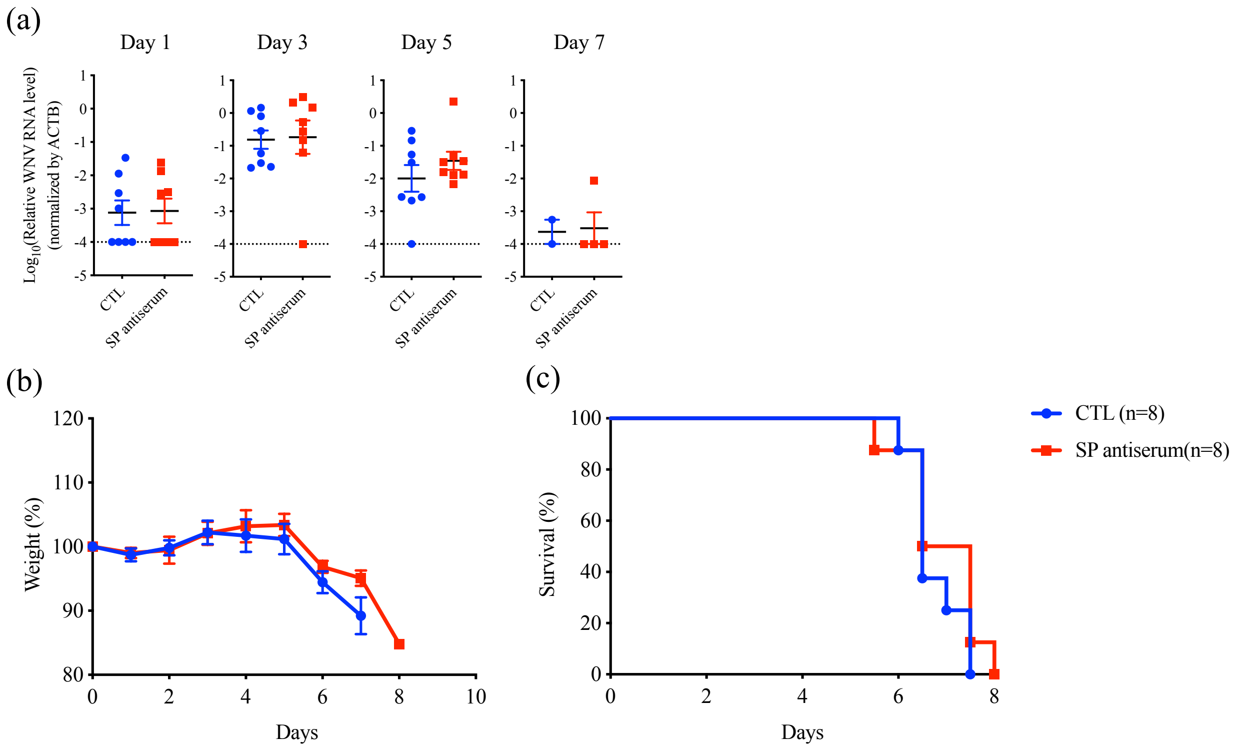

Supplementary Figure 1 Antiserum against SP was not protective against mosquito-borne West Nile virus infection.

(a) The virus levels in blood of mice treated with SP antiserum, fed by an infected mosquito. Blood was collected every other day for 7 days from mice fed on by WNV-infected mosquitoes and analyzed by qRT-PCR. WNV RNA levels were normalized to mouse *β* actin RNA levels. Mice immunized with naïve serum served as controls. Error bars represent mean ± SEM. Each data point represents one mouse. Normalized viral RNA levels were analyzed using one-tailed Wilcoxon–Mann–Whitney test.

(b) The weight of mice fed by an infected mosquito. Mice were monitored daily after WNV infection. Error bars represent mean ± SEM. Weight at each time point were compared using one-tailed Wilcoxon–Mann–Whitney test.

(c) Survival was assessed by a Gehan-Wilcoxon test (n=8/each group biologically independent samples pooled from two separate experiments).

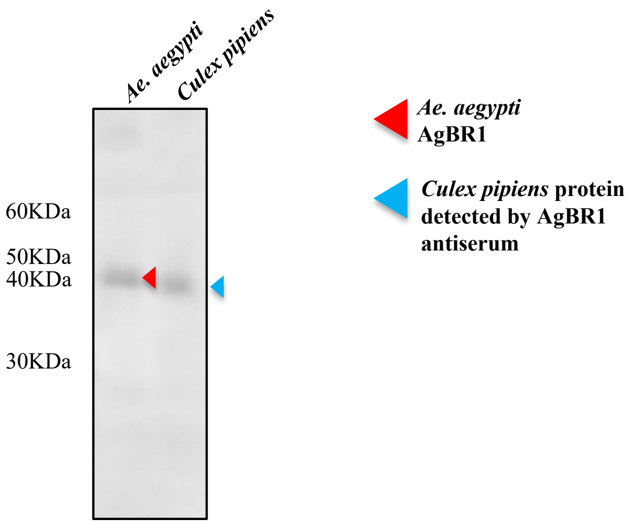

Supplementary Figure 2 Reactivity of AgBR1 serum against *Aedes aegypti* salivary glands and *Culex pipiens*.

Immunoblot of *Ae. aegypti* and *Culex pipiens* salivary glands. Blot was probed with rabbit AgBR1 antiserum.

Supplementary Fig. 2

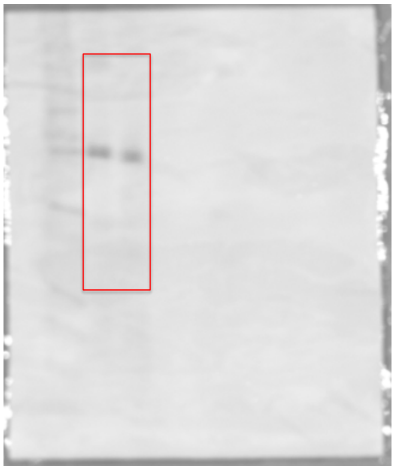

Supplementary Figure 3 The uncropped original blot.
